# Electrocardiogram Changes as an Independent Predictive Factor of Mortality in Patients with Acute Ischemic Stroke; a Cohort Study

**Published:** 2019-04-27

**Authors:** Payman Asadi, Seyyed Mahdi Zia Ziabari, Donya Naghshe Jahan, Arezoo Jafarian Yazdi

**Affiliations:** 1 Road Trauma Research Center, School of Medicine Guilan University of Medical Sciences, Rasht, Iran.

**Keywords:** Stroke, brain ischemia, patient outcome assessment, electrocardiography, prognosis, emergency service, hospital

## Abstract

**Introduction:**

Various factors such as age and severity of the stroke have been deemed connected with risk of mortality in patients with acute ischemic brain stroke. The present study was performed with the aim of evaluating the role of electrocardiogram (ECG) changes in predicting the outcome of these patients.

**Methods:**

In this cohort study, patients who had presented to the emergency department of a teaching hospital during 1 year and were diagnosed with acute ischemic stroke were evaluated. Demographic data and 12-lead ECG findings of the patients were gathered and their relationship with 1-year mortality was analyzed.

**Results:**

Finally, 546 stroke patients with the mean age of 69.5±12.7 (24 – 100) years were studied (53.3% female). 82.7% of the studied patients had at least one of the evaluated ECG abnormalities. The most common ECG findings included normal sinus rhythm (27.3%), inverted T wave (21.2%), sinus tachycardia (11.7%), atrial fibrillation (AF) (11.5%), and pathologic Q wave (9.9%). In the end, 117 (20.9%) patients died during the 1-year follow-up. Frequencies of non-sinus rhythm (p < 0.0001), inverted T wave (p = 0.0001), AF rhythm (p<0.0001), pathologic Q (p<0.0001), ST segment changes (p = 0.011), and atrioventricular (AV) node block (p = 0.007) were significantly higher in patients who died. ECG changes increased the odds of 1-year mortality of these patients 4 times (Odds ratio = 4.05 with 95% CI: 2.39 - 6.87; p < 0.0001). Additionally, age over 60 years and having a history of cardiac diseases increased the odds of mortality 6 (95% CI: 1.4 – 27.9) and 1.5 (95% CI: 0.9 – 2.1) times, respectively.

**Conclusion:**

Based on the findings of the present study, it seems that along with age and history of cardiac diseases, ECG changes can be considered as an independent predictive factor of mortality in patients with ischemic stroke.

## Introduction:

Stroke is one of the important causes of mortality and dysfunction, and can affect the quality of life of both patients and their relatives (1, 2). It is estimated that in the United States about 57.9 billion dollars is spent on stroke and its consequences each year (3). Every year, 5 million people die following stroke all over the world and at least one sixth of those who survive will have another stroke in the 5 following years. Two third of stroke cases happen in developing countries and 80% of all mortalities due to stroke also happen in these countries (4). Various factors such as age and severity of the stroke have been deemed connected with risk of mortality in these patients (5). Electrocardiography plays an important role in detecting a number of risk factor predicting stroke such as atrial fibrillation and left ventricular hypertrophy, which are components of Framingham stroke risk profile (6). On the other hand, brain stroke itself and increase in the intracranial pressure can lead to changes in electrocardiogram (ECG) even in the absence of history of cardiac underlying illnesses (7). Most common changes in ECG reported in patients with acute ischemic stroke include QT prolongation, T wave abnormalities, atrioventricular (AV) block, prominent U wave, and ST segment abnormalities (8, 9). Atrial fibrillation (AF) has also been commonly reported but most of the times it is not known whether AF leads to cardioembolic accidents, or it is secondary to the brain ischemia (10).

Some studies have shown that ECG changes can be considered as predictive factors for brain stroke outcomes and mortality due to it (11-13). However, studies are still ongoing on this subject. Therefore, the present study was performed with the aim of evaluating the role of ECG changes in predicting the outcome of patients presenting to the emergency department following acute ischemic stroke.

## Methods:

***Study design and setting***

In this cohort study, patients who had presented to the emergency department of Poursina Hospital, Rasht, Iran, during 1 year and were diagnosed with acute ischemic stroke were evaluated. Demographic data and 12-lead ECG findings of the patients were gathered and their relationship with 1-year outcome of the patients was analyzed. Protocol of the study was approved by the ethics committee of Guilan University of Medical Sciences. The researchers adhered to confidentiality of patients’ data.

***Participants***

All the patients presenting to the emergency department with diagnosis of acute ischemic stroke were included without considering any age or sex limitation. Patients’ medical profile being unavailable, being lost to follow-up, having history of dysrhythmia, and not giving consent for participation in the study were among the most important exclusion criteria of the study.

***Data gathering***

Demographic data, history of underlying illnesses, and the findings of 12-lead ECG were gathered after registration on their clinical profile. Diagnosis of ischemic stroke was done based on clinical findings and imaging, by an emergency medicine specialist and a neurologist. In addition, ECGs of all the patients were interpreted by an emergency medicine specialist. ECG changes evaluated in the present study included pathologic Q wave, AV node block, AF rhythm, inverted T wave, ST segment changes, left bundle branch block (LBBB), right bundle branch block (RBBB), sinus tachycardia, sinus bradycardia, atrial flutter, sinus arrest, sinus exit block, QT prolongation, and etc. Finally, all the patients included in the study were followed for 1 year from the time they were admitted to the emergency department using phone calls and cases with mortality related to brain stroke were recorded. A trained intern was responsible for data gathering and follow-up of the patients under direct supervision of the emergency medicine attend.

***Statistical analysis***

For analysis of data, SPSS 20 statistical software was used. The relationship between qualitative variables was evaluated using chi-square and Fisher’s exact test and the relationship between quantitative‌ variables was assessed via independent *t*-test. For multivariate analysis of mortality predictors, logistic regression model and backward LR method were applied. Odds ratio of independent factors in prediction of 1-year mortality were reported with 95% confidence interval (CI). Findings were reported as mean and standard deviation, or frequency and percentage. Significance level of the tests was considered to be p < 0.05 in this study.

## Results

***Baseline characteristics of the studied patients***

600 patients were included in the study, 54 cases were eliminated due to various reasons (36 cases were lost to follow-up and in 18 cases data were not recorded accurately). Finally, 546 stroke patients with the mean age of 69.5±12.7 (24 – 100) years were studied (53.3% female). 36.1% (197 cases) of the participants had a history of cardiac diseases, 63.9% (349 patients) had a history of hypertension, 32.6% (178 participants) had a history of diabetes, and 10.6% (58 cases) were smokers. 82.7% of the studied patients had at least one of the evaluated ECG abnormalities. The most common ECG findings of the studied patients included normal sinus rhythm (27.3%), inverted T wave (21.2%), sinus tachycardia (11.7%), atrial fibrillation (11.5%), and pathologic Q wave (9.9%). In the end, 117 (20.9%) patients died during the 1-year follow-up. 

**Table 1:** Comparison of ECG findings between ischemic stroke patients who died and those who survived

**ECG findings**	**Died **	**Survived **	**P value**
Normal sinus rhythm(NSR)	21 (10.5)	128 (37.0)	< 0.001
Inverted T wave	59 (29.5)	57 (16.5)	< 0.001
Sinus tachycardia	26 (13.0)	38 (11.0)	0.480
Atrial fibrillation (AF)	47 (23.5)	16 (4.6)	< 0.001
Pathologic Q wave	32 (16.0)	22 (6.4)	< 0.001
Sinus bradycardia	13 (6.5)	26 (7.5)	0.657
Poor R wave progression	13 (6.5)	23 (6.6)	0.947
Late transition	14 (7.0)	18 (5.2)	0.389
ST segment changes	18 (9.0)	13 (3.8)	0.011
Left ventricular hypertrophy	13 (6.5)	14 (4.0)	0.203
Left anterior superior fascicular block	7 (3.5)	17 (4.9)	0.438
Right bundle branch block (RBBB)	10 (5.0)	17 (4.9)	0.964
Left bundle branch block (LBBB)	8 (4.0)	10 (2.9)	0.484
Left atrial enlargement	5 (2.5)	16 (4.6)	0.214
Atrioventricular block	11 (5.5)	5 (1.4)	0.007
QT prolongation	7 (3.5)	6 (1.7)	0.192
Other	34 (44.7)	42 (55.3)	0.114

Data are presented as frequency (%).

**Figure 1:**
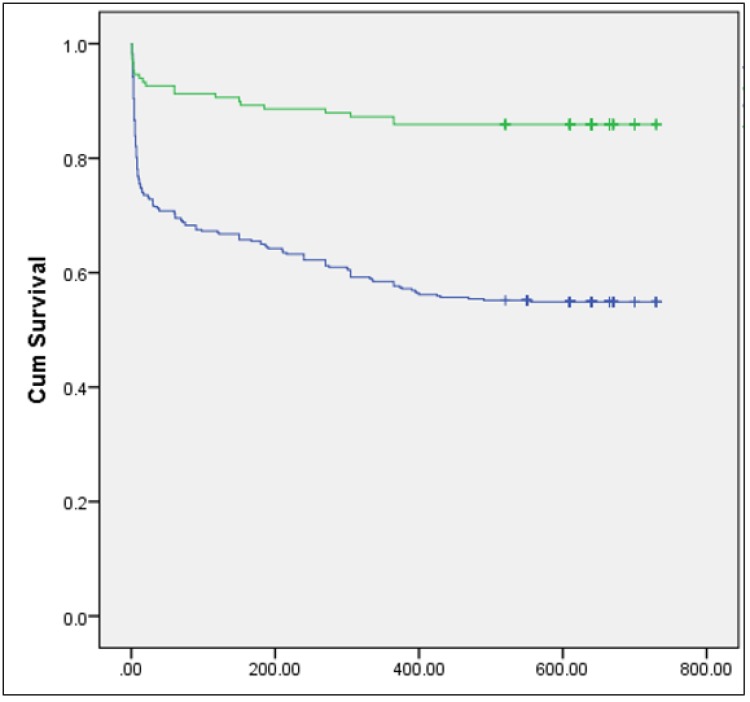
Survival rate of ischemic stroke patients based on presence (green line) or absence (blue line) of normal sinus rhythm. X axis= time (days), Y axis: cumulative survival

***Predictive factors of mortality***

On average, those who survived were about 10 years younger than those who died (65.9±12.6 versus 75.9±10.2, p<0.0001). In addition, there was a significant correlation between the history of cardiac diseases (p<0.0001) and history of hypertension (p = 0.01) with mortality. But there was no significant correlation between mortality and sex (p = 0.311), having diabetes (p = 0.779), or smoking (p = 0.920). ECG findings of the studied patients based on mortality status have been summarized in table 1. Frequencies of non-sinus rhythm (p < 0.0001), inverted T wave (p = 0.0001), AF rhythm (p<0.0001), pathologic Q (p<0.0001), ST segment changes (p = 0.011), and AV node block (p = 0.007) were significantly higher in patients who died.

The mortality rate of patients with abnormal and normal ECGs was 45.1% and 14.1%, respectively (p = 0.0001). This rate was 59.3% in patients with inverted T wave and 73.8% in those with AF rhythm.

Based on the logistic regression model with Backward LR method, ECG changes was found to be an independent predictor of mortality in patients with stroke, so that changes in ECG increased the odds of 1-year mortality of these patients 4 times (Odds ratio = 4.05 with 95% CI: 2.39 - 6.87; p < 0.0001). Based on this model, age over 60 years and having a history of cardiac diseases also increased the odds of mortality 6 (95% CI: 1.4 – 27.9) and 1.5 (95% CI: 0.9 – 2.1) times, respectively.

## Discussion:

Based on the findings of the present study, it seems that along with age and history of cardiac diseases, changes in ECG can be considered as an independent factor predicting 1-year mortality of the patients with ischemic stroke. Occurrence of ECG changes, increases the odds of 1-year mortality of stroke patients about 4 times.

In the present study, more than 80% of the patients had at least one change in ECG. Christensen et al. reported the rate of changes in ECG among patients with ischemic brain stroke to be about 60% (12). Koochaki et al. also reported the prevalence of ECG changes in patients with ischemic stroke to be 68.3% (14). In 2010, Van Bree et al. expressed that 81% of their studied patients in Netherlands had at least one abnormality in their ECG (15). Overall, considering the results of the present study and other studies, it can be concluded that ECG changes are very common among patients with stroke.

In the present study, the most common changes in ECGs of the patients were inverted T wave, AF rhythm, and pathologic Q wave, respectively. In different studies, findings regarding the most common ECG changes observed vary. In the study by Koochaki et al. inverted T wave and ST segment elevation/depression were the most common changes observed in ECG of the patients, both of which had a prevalence of 27.93%. After that, pathologic Q wave and LBBB were the most common with 18.44% and 17.88%, respectively (14). In the study by Maurits van Bree et al. in 2010 in Netherlands, the most common ECG changes among patients with brain stroke were QTC prolongation (36%), morphologic changes in ST-T (23%), sinus bradycardia (16%), and inverted T wave (16%) and, respectively (15). In a study by Marini et al. in 2005 in Italy, which evaluated AF and outcome of stroke, AF was observed in the beginning and during the acute phase in 24.6% of the patients with stroke (13), which is close to the rate of AF prevalence observed among the stroke patients in the present study.

Marini et al. in 2005 stated that the probability of 30-day and 1-year mortality in patients with stroke correlated with changes in ECG and age (13). Increase in mortality in older ages can be evaluated from different perspectives. First, older patients have less recovery power compared to younger patients and on the other hand, patients with older ages are more exposed to risk factors of stroke such as hyperlipidemia, hypertension, atherosclerosis, and other cardiovascular diseases that overall lead to an increased mortality in older patients (16-19). In addition, the rate of mortality was significantly higher in patients with a history of cardiovascular diseases in the present study, which is in line with the findings of previous studies (19).

Mortality rate was 14.1% among patients with stroke with normal ECG, while in stroke patients with abnormal ECG the rate increased to 45.1%. The rate of mortality in patients with inverted T wave as the most common change in ECG among patients was 59.3%. In the present study, the highest rate of mortality in stroke patients who had changes in ECG, belonged to those who had AF with 73.8%. Bozluolcay et al in 2003 in Turkey reported the rate of mortality in stroke patients with abnormal ECG to be 38.9% versus 15.2% in those who had a normal ECG (20). In the study by Marini et al. it was expressed that among patients with stroke, 32.5% of those who had AF died within 30 days and 49.5% died within a year; meanwhile, mortality rate for patients without AF was 16.2% and 27.1% during 30 days and 1 year, respectively (13), which is very different from the findings of the present study. The disagreement might be due to the differences in the studied populations and sampling methods of the studies.

In the study by Koochaki et al. in 2012, it was shown that ECG abnormalities are common in patients with acute ischemic stroke and cardiac evaluations should have predictive value (14). In the present study, after performing regression test, it was revealed that ECG changes in patients with stroke increase the odds of mortality about 4 times. Additionally, in a study by Goldstein it was reported that changes in ECG elevate the probability of mortality to 4.82 times (21), which is similar to the finding of the present study.

From the results of the present study and previous ones, it can be concluded that ECG changes, especially ST segment changes, inverted T wave, and AF, are very common in patients with ischemic stroke and are associated with increased mortality in these patients along with factors such as age and presence of cardiac diseases.

Of course for generalizing the results of this study, there is still need for more accurate studies by eliminating the limitations of the present and previous studies. 

***Limitations:***

In the present study, some of the potential factors affecting the outcome of patients with ischemic stroke, such as the size of the ischemic region, were not evaluated; it is suggested to assess imaging findings as one of the probable variables affecting outcome. In addition, in the present study, the time interval between manifestation of symptoms and presenting to the emergency department and undergoing electrocardiography has not been reported. However, it seems that this matter could affects development of complications such as increase in intracranial pressure and thus, the probability of developing the abnormal ECG findings caused by it. Another very important limitation is that it is not known whether observed changes in ECG are new or old. For a more accurate study, only patients that have evidence of a normal ECG before the stroke should be included in the study. However, despite all of these limitations, the findings of the study can present a relatively accurate estimation of the current status for planning future studies.

## Conclusion:

Based on the findings of the present study, it seems that along with age and history of cardiac diseases, changes in ECG can be considered as an independent factor predicting 1-year mortality of the patients with ischemic stroke. Occurrence of ECG changes following stroke, increases the odds of 1-year mortality about 4 times.
